# Intestinal Parasitic Infections and Associated Risk Factors among Food Handlers of Food and Drinking Establishments in Woldia Town, North-East Ethiopia: A Cross-Sectional Study

**DOI:** 10.1155/2023/2831175

**Published:** 2023-01-14

**Authors:** Daniel Getacher Feleke, Habtye Bisetegn, Getamesay Zewudu, Yohannes Alemu, Seifegebriel Teshome Feleke

**Affiliations:** ^1^Department of Microbiology, Immunology and Parasitology, College of Health Sciences, Addis Ababa University, Addis Ababa, Ethiopia; ^2^Department of Medical Laboratory Science, College of Medicine and Health Sciences, Wollo University, Dessie, Ethiopia

## Abstract

**Background:**

Food handlers should be screened periodically for intestinal parasitic infections, and they should be treated to reduce intestinal parasite transmission to consumers through contaminated foods and drinks. Therefore, this study aimed to assess the prevalence and associated risk factors of intestinal parasitic infection among food handlers in Woldia town, North-East Ethiopia.

**Method:**

A community-basedcross-sectional study was conducted among food handlers in Woldia town, North-East Ethiopia. A structured questionnaire was used to collect sociodemographic characteristics and intestinal parasite-associated risk factors. Microscopic examination of a stool sample was performed using wet-mount and formol-ether concentration techniques. Data were analyzed using SPSS version 20.0 statistical software packages. Bivariate and multivariate analyses were performed to investigate the association between intestinal parasitic infections and associated risk factors. In all comparisons, *P* value <0.05 was considered as statistically significant.

**Result:**

The overall prevalence of intestinal parasitic infection among food handlers in Woldia town was 14.3%. Six different intestinal parasites were detected. The majority of the parasites identified were helminthic infections 37/52 (71%). *Ascaris lumbricoides* was the most dominant parasite (7.7%), followed by *E. histolytica/dispar* (2.7%) and *G. lamblia* (1.4%). Multivariate logistic regression analysis showed that intestinal parasitic infection had a statistically significant association with food handlers' habits of hand washing without soap after latrine use (*P* < 0.01), swimming habit (*P*=0.03), and using a common knife (*P* < 0.01).

**Conclusion:**

This study revealed a relatively high prevalence of intestinal parasites among food handlers in Woldia town. Strict and standard hygienic and sanitary practices should be implemented by food handlers. Moreover, food handlers should be screened for intestinal parasitic infection, and health education should be given periodically.

## 1. Background

Intestinal parasitic infections affected an estimated 3.5 billion people and 450 million are ill and more than 200,000 people are died annually worldwide [1, 2]. *Ascaris lumbricoides and Giardia lamblia* infections have the highest rate of infection among all kinds of intestinal parasites [3]. Intestinal parasitic infections are transmitted directly or indirectly through contaminated food, water, fruits, and vegetables [2, 4]. They are highly endemic in developing countries due to poor socioeconomic conditions, poor hygiene and sanitation practice, and lack of safe and adequate water supply [5]. Furthermore, food handlers in developing countries are usually recruited into food and drinking establishments without being screened for intestinal parasites [6]. Asymptomatic food handlers infected with intestinal parasites considered as a source of infection for the community as they routinely practice their jobs without any clinical signs and symptoms [7, 8]. Food handlers' personal hygiene and sanitation conditions are the main sources of intestinal parasites infections worldwide [9].

In Ethiopia, intestinal parasitic infections are the major public health problems that causes of mortality and morbidity. The high prevalence of intestinal parasitic infection in Ethiopia could be due to poor environmental sanitation, overcrowding, poor socioeconomic status, irrigation, and low altitude. Moreover, eating and drinking from food service establishments is a common practice in Ethiopia [10]. This can increase the risk of intestinal parasitic infections from contaminated food and drinks due to unhygienic handling by food handlers. Therefore, assessment of intestinal parasites among symptomatic and asymptomatic food handlers employed in food and drink establishments and treating them with appropriate drugs has paramount importance to reduce and control the spread of intestinal parasitic infections. Therefore, apart from institution-based studies community-level assessment of intestinal parasitic infections and associated risk factors among food handlers in different localities is very important to reduce intestinal parasite transmission to the community and to sensitize food and drink establishments' regulatory bodies to enhance their follow-up to safeguard the community's health. Therefore, this study aimed to investigate the prevalence intestinal parasitic infections and associated factors among food handlers in Woldia town, North-East Ethiopia.

## 2. Methods

### 2.1. Study Area

This study was conducted in Woldia town, the capital of the North Wollo zone, located in the Amhara region. The town is 525 km far from Addis Ababa, the capital city of Ethiopia. It is found at an elevation of 2112 meters above sea level. The estimated population of Woldia town for 2021 was 98,911. The food and drink establishments in Woldia town include one international hotel, 150 hotels, restaurants, cafes, and 208 fast food houses.

### 2.2. Study Design and Period

This community-basedcross-sectional study was conducted in Woldia town from February 2019 to June 2019.

### 2.3. Eligibility Criteria

All food handlers in Woldia town food and drink establishment services were included in the study. However, food handlers with signs and symptoms of intestinal parasitic infections, diarrhoea, and fever and those who took any antiparasitic drugs at the time of the study or two weeks prior to the study were excluded.

### 2.4. Sample Size Determination and Sampling Technique

A single population proportion formula with the assumptions of a 95% confidence interval (*Zα*/2 = 1.96), 62.2% proportion [11], and 5% margin of error was used for sample size calculation. The final sample size was 364. Simple random sampling technique was used to recruit study participants. First, food and drink establishments were selected randomly from the total hotels, restaurants, cafes, fast food centres, and meat houses in Woldia town. The selected food establishments comprised one international hotel, 25 hotels, restaurants, and cafes, 9 meat houses, and 38 fast food centres. Then, study participants from each food establishment were selected proportionally based on the size of their food handlers using a simple random sampling technique.

### 2.5. Data Collection and Laboratory Investigation

A pretested structured questionnaire was used to collect sociodemographic characteristics and risk factors associated with intestinal parasitic infections. Data collection training was given to the data collectors before the start of the study. Stool specimen was collected from each study participant using a leak-proof and clean plastic container for parasitological examination. About 2 g of fresh stool was collected and examined microscopically using both the direct wet mount technique and the formol-ether concentration technique. Moreover, a stool sample was preserved with 10% formalin and transported to the Wollo University parasitology laboratory for formol-ether concentration technique procedures. Microscopic examination of sediment was performed by strictly following standard operating procedures (SOPs).

### 2.6. Data Analysis

Data were entered into “Epi Data 3.1” software and exported to SPSS version 20.0 statistical software packages for analysis. Bivariate and multivariate analyses were done to investigate the association between intestinal parasitic infections and associated risk factors. In all comparisons, *P* value <0.05 was considered as statistically significant.

### 2.7. Ethical Consideration

Ethical clearance was obtained from the Department of Medical Laboratory Science, College of Medicine and Health Sciences, Wollo University. Permission to conduct the study has also been obtained from the Woldia town health office. Written consent was obtained from each study participant. Intestinal parasite infected food handlers were treated with the appropriate antiparasitic drugs.

## 3. Results

### 3.1. Sociodemographic Characteristics

A total of 364 food handlers were involved in this study, with a 100% response rate. The mean age was 23.91 ± 4.023 years. The majority of the study participants were females (209 (57.4%)). Most of the food handlers (111 (30.5%)) had college and above education level. Furthermore, 32.1% of food handlers had less than one year food handling experience. Most of the food handlers 302 (90.9%) had access to clean latrine, and 62 (17.0%) of the study participants had habit of hand washing without soap after latrine use. Most, 300 (82.4%) of the food handlers had no formal medical certification (Table 1).

### 3.2. Prevalence of Intestinal Parasites among Food Handlers in Woldia Town

The overall prevalence of intestinal parasitic infection in Woldia town was 14.3% (52/364). Six different intestinal parasites were identified. The dominant intestinal parasite was *Ascaris lumbricoides* (7.7%), followed by *Entamoeba histolytica/dispar* (2.7%), *Giardia lamblia* (1.4%), and *Hymenolepis nana* (1.1%). The majority of parasites detected among food handlers were helminthic infections 37 (71%) (Table 2).

Bivariate analysis showed that the prevalence of intestinal parasitic infections had a statistically significant association with food handlers educational status, hand washing habit with soap after latrine visit, swimming habit, and knife use habit (*P* < 0.05). In multivariate analysis, food handlers who had habit of hand washing without soap after latrine use were more likely to be infected with intestinal parasites (AOR = 20.7, 95 CI: 2.03–4.20) compared to those who did not wash their hands with soap after latrine use. Furthermore, food handlers who had used a common knife during food preparation were more likely to be infected with intestinal parasites (AOR = 2.56, 95 CI 1.31–4.61) than those who used a private knife (Table 3).

## 4. Discussion

Intestinal parasites infections are among the most common infections transmitted through food contamination worldwide. Globally, an estimated that 3.5 billion people are affected and that 450 million people are ill as a result of intestinal parasitic infections, the majority of being children [11]. Food handlers infected with intestinal parasites, with or without signs and symptoms, could be a source of intestinal parasites in the community.

The present study showed that the prevalence of intestinal parasitic infection among food handlers in Woldia town was 14.3%. This finding was lower than reports from India (29.3%) [12], Pakistan (83.1%) [13], and Kenya (23.7%) [14]. Moreover, it was lower than reports of previous studies in different areas of Ethiopia. These studies were conducted in Addis Ababa University students' cafeteria (45.3%), Yebu town (44.1%), Arba Minch University students' cafeteria (36%), and Teda Health Centre (62.2%) [13–16]. This difference in prevalence might be due to variation in the study period, sociodemographic characteristics, personal hygiene practices of food handlers, food establishments sanitation, safe water supply, food handlers training about food hygiene, frequency of screening and treatment of food handlers, differences in knowledge of the transmission and prevention of intestinal parasites, and the methods of diagnosis used to detect intestinal parasites.

In this study, the prevalence of intestinal parasitic infections among food handlers was consistent with studies reported among food handlers in Aksum town, Northern Ethiopia (14.5%) [17], and Northern Iran (15.5%) [18]. This could be due to differences in the hygienic practice of food handlers and their awareness about hygienic food handling and the transmission, prevention, and control of intestinal parasites.

In the present study, the predominant parasite identified was *A. lumbricoides* (7.7%), followed by *E. histolytica/dispar* (2.7%) and *G. lamblia* (1.4%). The prevalence of *A. lumbricoides* in this study was relatively higher than similar previous studies reported from Ethiopia and Kenya [3, 13, 14]. The higher prevalence of *A. lumbricoides* might be associated with improper fecal disposal and unhygienic practice of food handlers during food preparation and serving.

On the other hand, the prevalence of *A. lumbricoides* was lower than previous reports from India (37.37%), Iran (55.8%), Yebu town, Ethiopia (17.8%) and Teda Health Centre, Ethiopia (23.2%) [12–14, 16]. In the present study, the prevalence of *G. lamblia* (1.4%) was lower than in previous studies from Ethiopia [13, 15, 17]. This might be due to the availability of clean and treated water in Woldia town. Most (92.3%) of the food handlers participated in this study had access to clean and treated water.

In the present study, food handlers who had a habit of hand washing without soap after latrine use were more likely to be infected with intestinal parasites (AOR = 20.7, 95 CI: 2.03–4.20) compared with those who did not wash their hands with soap after a latrine visit. This was in agreement with reports of previous studies from Ethiopia [13, 16]. However, this finding was not in agreement with previous reports from Swat, Iran [18], and Addis Ababa University students' cafeteria, Ethiopia [15]. Furthermore, food handlers who used a common knife during food preparation were 2.46 times more likely (AOR = 2.46, 95% CI 1.31–4.61) to be infected with intestinal parasites compared to those who used a private knife (*P* < 0.05). This was in agreement with reports from Swat, Iran [18] and Arba Minch University, Ethiopia [13]. This might be due to the fact that sharing knives during food preparation may transfer intestinal parasites from the unhygienic hands of food handlers to the foods and drinks they are preparing. As a result, intestinal parasitic infection could be increased among food handlers using common Knives.

In contrast to studies reported from Arba Minch University, Ethiopia [13] and Yebu town, Ethiopia [16], in this study, untrimmed finger nail was not statistically associated with intestinal parasitic infections. However, this finding was consistent with a study report from Addis Ababa University, Ethiopia [15]. Moreover, hand washing practice before meal had no significant association with intestinal parasitic infections. This was inconsistent with reports from Ethiopia [13, 16]. This might be due to the fact that in this study food handlers had a good hand washing practice before meal. Most of the food handlers (92.3%) participated in this study had a good habit of washing their hands before meal.

## 5. Conclusion

This study revealed that the prevalence of intestinal parasites among food handlers in Woldia town was relatively high. Risk factors such as hand washing habits after toileting without soap, the habit of using a common knife, and swimming habits had a statistically significant association with intestinal parasitic infection. Symptomatic and asymptomatic food handlers infected with intestinal parasites could be an important source of infection to the community due to the fecal-oral transmission of intestinal parasites. Therefore, strict and standard hygienic and sanitary practices should be followed by food handlers to improve personal hygiene. Moreover, food handlers should be screened for parasitic infections periodically. Health education should also be provided for food handlers on personal hygiene, environmental sanitation, food hygiene, intestinal parasites transmission, prevention, and control.

## Figures and Tables

**Table 1:** Sociodemographic characteristics and risk factors associated with intestinal parasitic infections among food handlers in Woldia town, North-East Ethiopia.

Variables	Category	Number (%)
Sex	Male	155 (42.6)
	Female	209 (57.4)

Age	<20	132 (36.3)
	20–35	203 (55.8)
	>35	29 (8.0)

Educational status	Illiterate	68 (18.7)
	Primary education	97 (26.6)
	Secondary education	88 (24.2)
	College and above	111 (30.5)

Occupation	Chef	149 (40.9)
	Dish washer	47 (12.9)
	Waiter	148 (40.7)
	Barista	12 (3.3)
	Butcher	8 (2.2)

Service year	<1 year	117 (32.1)
	1-2	95 (26.1)
	3–5	111 (30.5)
	>5	41 (11.3)

Nail trimmed	Yes	266 (73.1)
	No	98 (26.9)

Have access to clean latrine	Yes	302 (90.9)
	No	33 (9.1)

Hand washing habit with soap after latrine	Yes	302 (83.0)
	No	62 (17.0)

Knife use habit	Common	82 (22.5)
	Private	282 (77.5)

Health education on food handling	Yes	146 (40.1)
	No	218 (59.9)

Safe water drink access	Yes	336 (92.3)
	No	28 (7.7)

Had medical certificate	Yes	64 (17.6)
	No	300 (82.4)

**Table 2:** Prevalence of intestinal parasite among food handlers in Woldia town, North-East Ethiopia.

Intestinal parasites detected	Number (%)
*Ascaris lumbricoides*	28 (7.7)
*Entamoeba histolytica/dispar*	10 (2.7)
*Giardia lamblia*	5 (1.4)
Hook worm	3 (0.8)
*Enterobius vermicularis*	2 (0.5)
*Hymenolepis nana*	4 (1.1)
Total	52 (14.3)

**Table 3:** Intestinal parasitic infections and associated risk factors among food handlers at Woldia town, North-EastEthiopia.

Variables	Category	Intestinal parasitic infections		COR (95% CI)	*P* values	AOR (95% CI)	*P* values
		Positive (*N* (%))	Negative (*N* (%))				
Educational status	Illiterate	15 (22.1)	53 (77.9)	2.88 (1.22–6.83)	**0.01**	1.18 (0.43–3.24)	0.75
	Primary education	12 (12.4)	85 (87.6)	1.10 (0.46–2.46)	0.88		
	Secondary education	12 (13.6)	88 (86.4)	1.19 (0.51–2.76)	0.68		
	College and above	13 (11.7)	111 (88.3)	1		1	

Finger nail status	Trimmed	40 (15)	226 (85)	1			
	Untrimmed	12 (12.2)	86 (87.8)	1.27 (0.64–2.53)	0.50		

Hand washing after latrine with soap	Yes	24 (7.9)	278 (92.1)	1		1	
	No	28 (45.2)	34 (54.8)	2.11 (0.10–0.20)	**<0.01**	2.07 (2.03–4.20)	**<0.01**

Hand washing habit before meal	Yes	45 (13.4)	291 (86.6)	1			
	No	7 (25.0)	21 (75.0)	0.46 (0.19–1.15)	0.09		

Swimming habit	Yes	9 (9.0)	91 (91.0)	1		1	
	No	43 (16.3)	221 (83.7)	0.44 (0.20–0.96)	**0.04**	0.46 (0.21–1.01)	**0.03**

Knife use habit	Common	20 (24.4)	62 (75.6)	2.52 (1.35–4.70)		2.46 (1.31–4.61)	
	Private	32 (11.3)	250 (88.7)	1	**<0.01**	1	**<0.01**

This is just to show those bold values are statistically significant.

## Data Availability

All data underlying the findings are fully available without restriction. All relevant data are within the manuscript.
